# A novel entity of HIPK2::YAP1 pulmonary fibromatosis

**DOI:** 10.1186/s12890-024-03026-5

**Published:** 2024-05-07

**Authors:** Yuqiang Liu, Meng Liang, Kai Chen, Lucas Wang, Yaxian Yang, Qi Li, Bin Lian, Tongxu Zhuo, Jian Huang

**Affiliations:** 1 Department of Proctology, Honliv Hospital, Xinxiang, 453400 Henan Province China; 2 Department of Academic Affairs, Xinxiang Medical University, Xinxiang, 453003 Henan Province China; 3 Department of Respiratory Medicine, Yongcheng People Hospital, Yongcheng, 476600 Henan Province China; 4 Precision Medicine Center, Guangzhou Huayin Health Medical Group Co., Ltd, No.33 Binhe Road, Huangpu District, Guangzhou, 510700 Guangdong Province China; 5 Department of Pathological Diagnosis and Research Center, the Affiliated Hospital of Guangdong Medical University, No.57 South Renmin Avenue, Xiashan District, Zhanjiang, 524001 Guangdong Province China

**Keywords:** Pulmonary fibromatosis, Heterogeneity, HIPK2-YAP1

## Abstract

**Background:**

Pulmonary fibromatosis (PF) is a specific variant of fibromatosis, which is rarely reported occurring in the lung. PF with *HIPK2-YAP1* fusion was a novel entity.

**Case presentation:**

In this report, a 66-year-old male with PF had been smoking over 40 years. Multiple cords and small nodules in both lungs had been detected in a health examination two years earlier at our hospital. But approximately twofold enlarged in the lingual segment of the upper lobe in the left lung were disclosed in this year. Immunohistochemical analysis demonstrated that the vimentin and β-Catenin were positive in the largest nodule. After underwent a DNA/RNA panel next-generation sequencing (NGS), missense mutations and *HIPK2-YAP1* fusion were found in this sample. Ultimately, the case diagnosis as PF with *HIPK2-YAP1* fusion after multidisciplinary treatment. Currently, the patient is doing well and recurrence-free at 14 months post-surgery.

**Conclusions:**

It’s difficult for patients with complex morphology to make accurate diagnosis solely based on morphology and immunohistochemistry. But molecular detection is an effective method for further determining pathological subtypes.

**Supplementary Information:**

The online version contains supplementary material available at 10.1186/s12890-024-03026-5.

## Background

Fibromatosis, first described by Stout et al. [1], is classified as benign in histological, but with the character of local invasiveness and local recurrence. Fibromatosis usually originating in intermuscular location, and identified at a median age in the 30 s.

Fibromatosis, also known as desmoids tumor, is classified into superficial and deep [2, 3]. Fibromatosis may involve essentially any location such as head and neck region, breast, colon, pelvic cavity, etc. [4]. Pulmonary fibromatosis (PF) is a specific variant of fibromatosis, which is rarely reported in the literature as occurring in the lung. Like other classifications of fibromatosis, PF is a widespread, locally aggressive fibroblastic tumor that occasionally recurs but never metastasizes [5]. Here we report a case of PF with *HIPK2-YAP1* fusion, the patient underwent thoracoscopic surgery and the resected specimen identified by histopathological, clinicopathological, immunohistochemical (IHC) and the genetics feature analysis.

## Case presentation

**Fig. 1:**
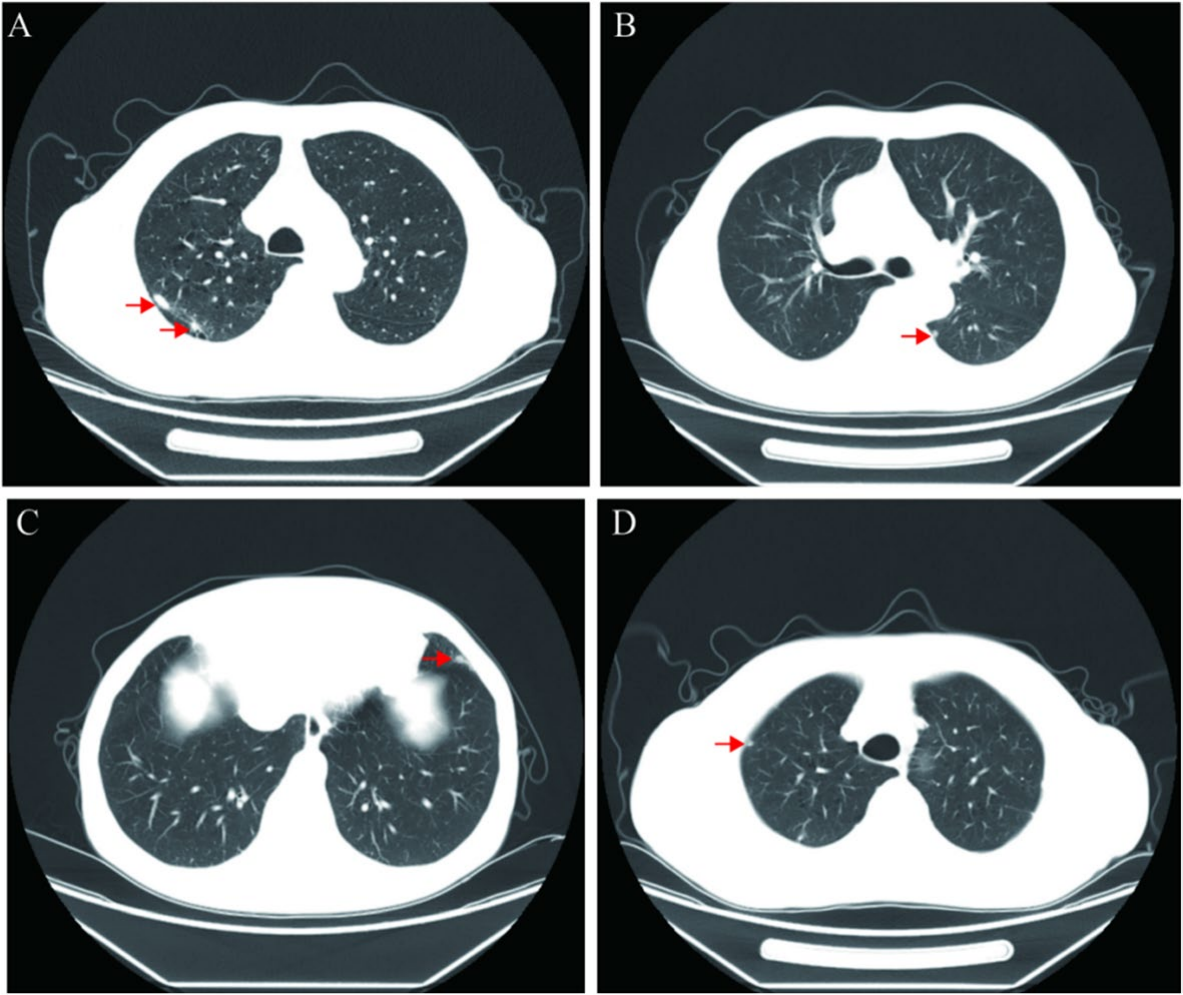
Enhanced computed tomography (CT) results of lung lobe: **A** 5.0 × 4.0 × 1.0 cm nodule was detected in the post-apical segment of the left upper lobe,1.5 × 1.0 × 1.0 cm nodule was detected in theapical segment of the left upper lobe; **B**-**D**Three tiny lung nodules(benign nodule with a measured diameter of 0.2–0.9 cm) detected in the lung and other parts of the right lung

A 66-year-old man with asymptomatic 40 years-smoking history was admitted to hospital due to multiple lung nodules detected in routine health checkup had roundly doubled in size in two years. Followed computed tomography (CT) imaging showed multiple small cystic translucent shadows with clear borders in both lungs. Besides, multiple small solid nodular shadows with clear borders were found in the upper lobe of both lungs and the lower lobe of the left lung. The largest nodules were found in the apical posterior segment of the upper lobe of the left lung with the size of about 5 mm × 4 mm × 1 mm (Fig. 1). Lymph node enlargement in the mediastinum was not detected. Scattered striated foci with clear borders were also seen in both lungs.

**Fig. 2:**
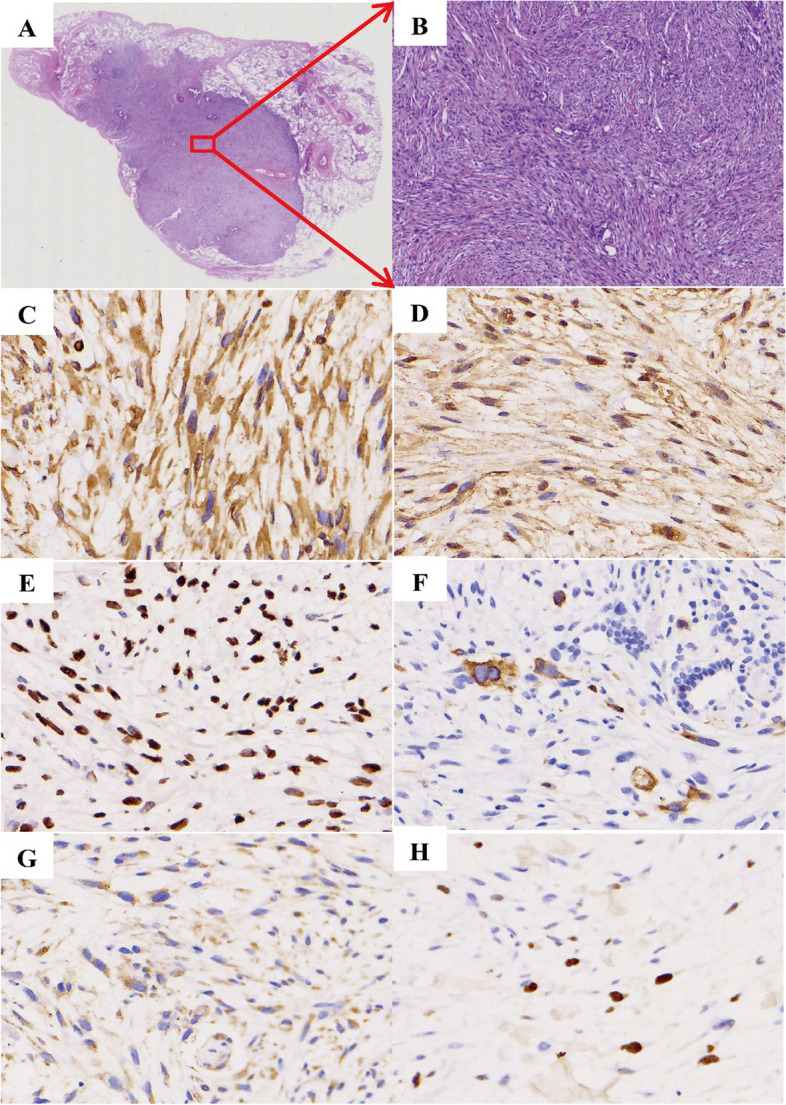
Histopathological features: **A** Overall the histopathology. **B** (HE-40×) Lung nodule biopsy revealing spindle-shaped cells within low malignant tendency. (C-H). IHC markers staining showed vimentin( +), β-Catenin( +), P53( +), NF(-), STAT-6 (-) and Ki-67 proliferation index in most of the tumor areas was around 10%

The patient underwent lung nodule removal surgery. Postoperative pathological examination suggested the lingual segment of the upper lobe left lung were nodal spindle cell tumor, consistent with low-grade malignancy of mesenchymal origin. The tumor cells exhibited bundled or woven pattern, with clear demarcation between the tumor and the surrounding lung tissue. The tumor cells were uniformly shuttle-shaped and of consistent size. While most areas showed no significant heterogeneity in the morphology of the tumor cells, some areas exhibited active growth with occasional nuclear fission images, indicating certain heterogeneity in the nuclei (Fig. 2A-B).

Other nodules shown proliferating spindle cells and collagen fibers, which were consistent with benign lesions. All nodules were close to the pleura but did not break through the pleura, and morphology was mainly characterized by proliferating spindle cells, collagen fibers and carbon deposits (Fig. S1 A-D).

Immunohistochemical of the upper lobe of the left lung (the largest nodule) results shown that the spindle cells stained positively for vimentin, β-Catenin and P53, negatively for NF and STAT-6. The percentage of Ki-67 positive nuclei was around 10% (Fig. 2C-H).

Genome sequencing was conducted using NGS (DNA-based 600 genes panel and RNA-based 86 fusion genes panel, panel lists were shown in Supplementary Tables 1 and 2) to make a precise diagnosis of the largest nodule. RNA-based NGS results showed a *HIPK2-YAP1* fusion (see detail fusion model in Supplementary Fig. 2). DNA-based NGS results shown 33.51% mutation abundance of *TP53* and 19.33% mutation abundance of *ARID1B*, which are considered as tumorigenic alterations.

The patient was finally diagnosed as PF with *HIPK2-YAP1* fusion mutations based on a multidisciplinary team discussion, which involved comprehensive consideration of past history, clinical manifestations, radiographic, histopathological, IHC and molecular pathology results. The patient is doing well without any signs of recurrence 14 months after surgery.

## Discussion and conclusions

As a rare benign pulmonary lesion, PF with *HIPK2-YAP1* fusion has not been reported previously. Histopathological and molecular features supply evidence for the final diagnosis. The tumor cells of fibromatosis have been reported to be positive for SMA, desmin, calponin, and estrogen receptor β, but negative for CD34. At molecular levels, fibromatosis sporadically harbors *CTNNB1* mutation, which encodes β-catenin protein [6]. In this case, *CTNNB1* mutation was not detected. It is possible that PF involve the CTNNB1 independent signaling pathway, but further confirmation is needed.

Although the function of the fusion variant between *HIPK2* and *YAP1* in the organism is not known, previous studies have shown that *HIPK2* expression was decreased in mouse model of pulmonary fibrosis, accompanied by the massive proliferation of alveolar collagen fibers with altered alveolar structure [7, 8]. This study [8] also suggested that low expression of *HIPK2* facilitated the proliferation and migration of mouse lung fibroblasts, inhibited apoptosis, and advanced the expression of mesenchymal markers and β-catenin. Similar view of was identified in human fibroblasts cell model [9] and anti-fibrotic therapy by targeting *HIPK2* may be a potentially effective therapeutic option. *HIPK2* is also reported as a tumor suppressor, because it’s involved in DNA damage repairment and apoptosis induction [10, 11]. In additionally, HIPK2 is considered as a caretaker tumor-suppressor, its inactivation increases tumorigenicity [12]. Even downregulation and mutations of HIPK2 are associated with poor prognosis in many types of human tumors [13].

In this case, *HIPK2-YAP1* fusion involving exon 1 of *HIPK2* and exons 3–9 of *YAP1*. This fusion disrupts the structure of HIPK2, which may lead to a partial or complete loss of function of the HIPK2 protein, allowing for an over proliferation of collagen fibers in epithelial cells such as lung fibroblasts, resulting in massive fibrosis of the patient's lung tissue, which in turn leads to fibromatosis. Consequently, further research is needed to illustrate its clinicopathological characteristics.

The diagnosis based on pathological morphology is the foundation of disease diagnosis, and molecular diagnosis is an important tool for accurate classification of diseases, which may bring more treatment opportunities for patients.

### Supplementary Information

**Supplementary Material 1. **

**Supplementary Material 2. **

## Data Availability

The data that support this case report are available from the corresponding author on reasonable request.

## Abbreviations

PF: Pulmonary fibromatosis
NGS: Next-generation sequencing
IHC: Immunohistochemical
CT: Computed tomography
