# Supplementary material for: A novel entity of HIPK2::YAP1 pulmonary fibromatosis
Source: BMC Pulm Med. 2024 May 7;24:223. doi: 10.1186/s12890-024-03026-5 (PMC11075317; doi:10.1186/s12890-024-03026-5)
Supplement: Supplementary file 1 — Supplementary Material 1. [file 12890_2024_3026_MOESM1_ESM.docx]

Supplementary Table 1. 600 genes panel list

| SNV、INDEL and CNV related genes | | | | | | |
| --- | --- | --- | --- | --- | --- | --- |
| A | | | | | | |
| *ABCB1* | *ABL1* | *ABL2* | *ACVR1* | *ACVR1B* | *AIM1* | *AKT1* |
| *AKT2* | *AKT3* | *ALK* | *ALOX12B* | *ANKRD11* | *ANKRD26* | *APC* |
| *AR* | *ARAF* | *ARFRP1* | *ARID1A* | *ARID1B* | *ARID2* | *ARID5B* |
| *ASXL1* | *ASXL2* | *ASXL3* | *ATG2B* | *ATG5* | *ATM* | *ATR* |
| *ATRX* | *AURKA* | *AURKB* | *AXIN1* | *AXIN2* | *AXL* |  |
| B | | | | | | |
| *B2M* | *BAP1* | *BARD1* | *BBC3* | *BCL10* | *BCL11B* | *BCL2* |
| *BCL2L1* | *BCL2L11* | *BCL2L2* | *BCL6* | *BCOR* | *BCORL1* | *BCR* |
| *BIRC3* | *BLM* | *BMPR1A* | *BRAF* | *BRCA1* | *BRCA2* | *BRD4* |
| *BRIP1* | *BTG1* | *BTK* |  |  |  |  |
| C | | | | | | |
| *C11ORF30* | *C11orf95* | *C8orf34* | *CALR* | *CARD11* | *CASP8* | *CBFB* |
| *CBL* | *CBLB* | *CBLC* | *CBR3* | *CCND1* | *CCND2* | *CCND3* |
| *CCNE1* | *CD274* | *CD276* | *CD28* | *CD3EAP* | *CD58* | *CD74* |
| *CD79A* | *CD79B* | *CDA* | *CDC73* | *CDH1* | *CDK12* | *CDK4* |
| *CDK6* | *CDK8* | *CDKN1A* | *CDKN1B* | *CDKN2A* | *CDKN2B* | *CDKN2C* |
| *CEBPA* | *CENPA* | *CHD1* | *CHD2* | *CHD4* | *CHD8* | *CHEK1* |
| *CHEK2* | *CIC* | *CIITA* | *CREBBP* | *CRKL* | *CRLF2* | *CSF1R* |
| *CSF3R* | *CSMD1* | *CSMD3* | *CTCF* | *CTLA4* | *CTNNA1* | *CTNNB1* |
| *CUL3* | *CUL4B* | *CUX1* | *CXCR4* | *CYLD* | *CYP19A1* | *CYP2D6* |
| *CYP2D7P* | *CYP4F3* |  |  |  |  |  |
| D | | | | | | |
| *DAXX* | *DCUN1D1* | *DDR2* | *DDX3X* | *DDX41* | *DHFR* | *DHX15* |
| *DICER1* | *DIS3* | *DNAJB1* | *DNM2* | *DNMT1* | *DNMT3A* | *DNMT3B* |
| *DOT1L* | *DPYD* | *DTX1* | *DUSP22* | *DYNC2H1* |  |  |
| E | | | | | | |
| *E2F3* | *EED* | *EGFL7* | *EGFR* | *EIF1AX* | *EIF4A2* | *EIF4E* |
| *EML4* | *EMSY* | *EP300* | *EPCAM* | *EPHA2* | *EPHA3* | *EPHA5* |
| *EPHA7* | *EPHB1* | *ERBB2* | *ERBB3* | *ERBB4* | *ERCC1* | *ERCC2* |
| *ERCC3* | *ERCC4* | *ERCC5* | *ERG* | *ERRFI1* | *ESR1* | *ETNK1* |
| *ETS1* | *ETV1* | *ETV4* | *ETV5* | *ETV6* | *EWSR1* | *EZH2* |
| F | | | | | | |
| *FAM175A* | *FAM46C* | *FANCA* | *FANCC* | *FANCD2* | *FANCE* | *FANCF* |
| *FANCG* | *FANCI* | *FANCL* | *FAS* | *FAT1* | *FBXW7* | *FGF10* |
| *FGF14* | *FGF19* | *FGF23* | *FGF3* | *FGF4* | *FGF6* | *FGF7* |
| *FGFR1* | *FGFR2* | *FGFR3* | *FGFR4* | *FH* | *FLCN* | *FLI1* |
| *FLT1* | *FLT3* | *FLT4* | *FOLR3* | *FOXA1* | *FOXL2* | *FOXO1* |
| *FOXO3* | *FOXP1* | *FRG1B* | *FRS2* | *FUBP1* | *FYN* |  |
| G | | | | | | |
| *GABRA6* | *GALNT12* | *GATA1* | *GATA2* | *GATA3* | *GATA4* | *GATA6* |
| *GID4* | *GLI1* | *GNA11* | *GNA13* | *GNAQ* | *GNAS* | *GPS2* |
| *GREM1* | *GRIN2A* | *GRM3* | *GSK3B* | *GSKIP* | *GSTM1* | *GSTP1* |
| *GSTT1* |  |  |  |  |  |  |
| H | | | | | | |
| *H3F3A* | *H3F3B* | *H3F3C* | *HDAC1* | *HDAC2* | *HGF* | *HIST1H1C* |
| *HIST1H2BD* | *HIST1H3A* | *HIST1H3B* | *HIST1H3C* | *HIST1H3D* | *HIST1H3E* | *HIST1H3F* |
| *HIST1H3G* | *HIST1H3H* | *HIST1H3I* | *HIST1H3J* | *HIST2H3C* | *HIST2H3D* | *HIST3H3* |
| *HLA-A* | *HLA-B* | *HNF1A* | *HOXB13* | *HRAS* | *HSD3B1* | *HSP90AA1* |
| I | | | | | | |
| *ICOSLG* | *ID3* | *IDH1* | *IDH2* | *IFNGR1* | *IGF1* | *IGF1R* |
| *IGF2* | *IGF2BP3* | *IKBKE* | *IKZF1* | *IL10* | *IL7R* | *INHA* |
| *INHBA* | *INPP4A* | *INPP4B* | *INSR* | *IRF1* | *IRF2* | *IRF4* |
| *IRS1* | *IRS2* | *ITK* | *ITPKB* |  |  |  |
| J | | | | | | |
| *JAK1* | *JAK2* | *JAK3* | *JUN* |  |  |  |
| K | | | | | | |
| *KDM5A* | *KDM5C* | *KDM6A* | *KDR* | *KEAP1* | *KEL* | *KIF5B* |
| *KIF6* | *KIR2DL4* | *KIR3DL2* | *KIT* | *KLF2* | *KLF4* | *KLHL6* |
| *KLRC1* | *KLRC2* | *KLRK1* | *KMT2A* | *KMT2B* | *KMT2C* | *KMT2D* |
| *KRAS* |  |  |  |  |  |  |
| L | | | | | | |
| *LATS1* | *LATS2* | *LEF1* | *LMO1* | *LRP1B* | *LYN* | *LZTR1* |
| M | | | | | | |
| *MAGI2* | *MALT1* | *MAP2K1* | *MAP2K2* | *MAP2K4* | *MAP3K1* | *MAP3K13* |
| *MAP3K14* | *MAPK1* | *MAPK3* | *MAX* | *MCL1* | *MDC1* | *MDM2* |
| *MDM4* | *MED12* | *MEF2B* | *MEN1* | *MET* | *MFHAS1* | *MGA* |
| *MITF* | *MLH1* | *MLLT3* | *MPL* | *MRE11A* | *MSH2* | *MSH3* |
| *MSH6* | *MST1* | *MST1R* | *MTHFR* | *MTOR* | *MUTYH* | *MYB* |
| *MYC* | *MYCL1* | *MYCN* | *MYD88* | *MYOD1* |  |  |
| N | | | | | | |
| *NBN* | *NCOA3* | *NCOR1* | *NEGR1* | *NF1* | *NF2* | *NFE2L2* |
| *NFKBIA* | *NKX2-1* | *NKX3-1* | *NONO* | *NOTCH1* | *NOTCH2* | *NOTCH3* |
| *NOTCH4* | *NPM1* | *NQO1* | *NR4A3* | *NRAS* | *NRG1* | *NSD1* |
| *NTHL1* | *NTRK1* | *NTRK2* | *NTRK3* | *NUP93* | *NUTM1* |  |
| P | | | | | | |
| *PAK1* | *PAK3* | *PAK7* | *PALB2* | *PARK2* | *PARP1* | *PAX3* |
| *PAX5* | *PAX7* | *PAX8* | *PBRM1* | *PDCD1* | *PDCD1LG2* | *PDGFRA* |
| *PDGFRB* | *PDK1* | *PDPK1* | *PGR* | *PHF6* | *PHOX2B* | *PIK3C2B* |
| *PIK3C2G* | *PIK3C3* | *PIK3CA* | *PIK3CB* | *PIK3CD* | *PIK3CG* | *PIK3R1* |
| *PIK3R2* | *PIK3R3* | *PIM1* | *PLCG2* | *PLK2* | *PMAIP1* | *PML* |
| *PMS1* | *PMS2* | *PNRC1* | *POLD1* | *POLE* | *PPARG* | *PPM1D* |
| *PPP2R1A* | *PPP2R2A* | *PPP6C* | *PRDM1* | *PREX2* | *PRKAR1A* | *PRKCI* |
| *PRKDC* | *PRPF8* | *PRSS8* | *PTCH1* | *PTEN* | *PTPN11* | *PTPRD* |
| *PTPRS* | *PTPRT* |  |  |  |  |  |
| Q | | | | | | |
| *QKI* |  |  |  |  |  |  |
| R | | | | | | |
| *RAB35* | *RAC1* | *RAD21* | *RAD50* | *RAD51* | *RAD51B* | *RAD51C* |
| *RAD51D* | *RAD52* | *RAD54L* | *RAF1* | *RANBP2* | *RARA* | *RASA1* |
| *RB1* | *RBM10* | *RECQL4* | *REL* | *RELA* | *RET* | *RFWD2* |
| *RHEB* | *RHOA* | *RICTOR* | *RIT1* | *RNF43* | *ROS1* | *RPS6KA4* |
| *RPS6KB1* | *RPS6KB2* | *RPTOR* | *RSPO2* | *RUNX1* | *RUNX1T1* | *RYBP* |
| S | | | | | | |
| *SAMD9* | *SAMD9L* | *SDHA* | *SDHAF2* | *SDHB* | *SDHC* | *SDHD* |
| *SETBP1* | *SETD2* | *SF1* | *SF3A1* | *SF3B1* | *SGK1* | *SH2B3* |
| *SH2D1A* | *SHANK2* | *SHQ1* | *SLC19A1* | *SLIT2* | *SLX4* | *SMAD2* |
| *SMAD3* | *SMAD4* | *SMARCA4* | *SMARCB1* | *SMARCD1* | *SMC1A* | *SMC3* |
| *SMO* | *SNCAIP* | *SOCS1* | *SOD2* | *SOX10* | *SOX17* | *SOX2* |
| *SOX9* | *SPEN* | *SPI1* | *SPOP* | *SPTA1* | *SRC* | *SRP72* |
| *SRSF2* | *SRY* | *STAG2* | *STAT3* | *STAT4* | *STAT5A* | *STAT5B* |
| *STAT6* | *STK11* | *STK40* | *SUFU* | *SUZ12* | *SYK* |  |
| T | | | | | | |
| *TBX21* | *TBX3* | *TCEB1* | *TCF12* | *TCF3* | *TCF7L2* | *TEK* |
| *TERC* | *TERT* | *TET1* | *TET2* | *TFE3* | *TGFBR1* | *TGFBR2* |
| *THADA* | *TLX3* | *TMEM127* | *TMPRSS2* | *TNFAIP3* | *TNFRSF14* | *TOP1* |
| *TOP2A* | *TP53* | *TP63* | *TP73* | *TPMT* | *TRAF2* | *TRAF3* |
| *TRAF7* | *TSC1* | *TSC2* | *TSHR* | *TTK* | *TYMS* |  |
| U | | | | | | |
| *U2AF1* | *UGT1A1* | *UMPS* | *USP7* | *USP9X* |  |  |
| V | | | | | | |
| *VEGFA* | *VEGFB* | *VHL* | *VTCN1* |  |  |  |
| W | | | | | | |
| *WHSC1* | *WHSC1L1* | *WISP3* | *WT1* |  |  |  |
| X | | | | | | |
| *XPC* | *XPO1* | *XRCC1* | *XRCC2* | *XRCC3* |  |  |
| Y | | | | | | |
| *YAP1* |  |  |  |  |  |  |
| Z | | | | | | |
| *ZAP70* | *ZBTB7A* | *ZNF217* | *ZNF703* | *ZRSR2* |  |  |

Supplementary Table 2. 86 genes panel list

| **Soft tissue sarcoma related fusion gene** | | | | | | |
| --- | --- | --- | --- | --- | --- | --- |
| *ABL1* | *ALK* | *ASPSCR1* | *ATF1* | *ATIC* | *BCOR* | *BRAF* |
| *CAMTA1* | *CARS1* | *CCNB3* | *CIC* | *CITED2* | *CLTC* | *COL1A1* |
| *COL6A3* | *CREB1* | *CREB3L1* | *CREB3L2* | *CSF1* | *DDIT3* | *DUX4* |
| *EMILIN2* | *ERG* | *ETV1* | *ETV4* | *ETV6* | *EWSR1* | *FEV* |
| *FGFR1* | *FGFR2* | *FGFR3* | *FLI1* | *FOS* | *FOSB* | *FOXO1* |
| *FOXO3* | *FOXO4* | *FUS* | *GLI1* | *HEY1* | *INO80D* | *KMT2A* |
| *MAML2* | *MBNL1* | *MEIS1* | *NAB2* | *NCOA1* | *NCOA2* | *NR4A3* |
| *NTRK1* | *NTRK2* | *NTRK3* | *NUTM2A* | *NUTM2B* | *OGT* | *PATZ1* |
| *PAX3* | *PAX7* | *PDGFB* | *PDGFD* | *RAF1* | *RANBP2* | *RET* |
| *ROS1* | *SEPTIN7* | *SMAD3* | *SRF* | *SS18* | *SSX1* | *SSX2* |
| *SSX4* | *STAT6* | *TAF15* | *TCF12* | *TFE3* | *TFG* | *TPM3* |
| *TPM4* | *VGLL2* | *VIM* | *WT1* | *WWTR1* | *YAP1* | *YWHAE* |
| *ZC3H7B* | *ZFTA* |  |  |  |  |  |
