# Supplementary material for: A novel entity of HIPK2::YAP1 pulmonary fibromatosis
Source: BMC Pulm Med. 2024 May 7;24:223. doi: 10.1186/s12890-024-03026-5 (PMC11075317; doi:10.1186/s12890-024-03026-5)
Supplement: Supplementary file 2 — Supplementary Material 2. [file 12890_2024_3026_MOESM2_ESM.docx]

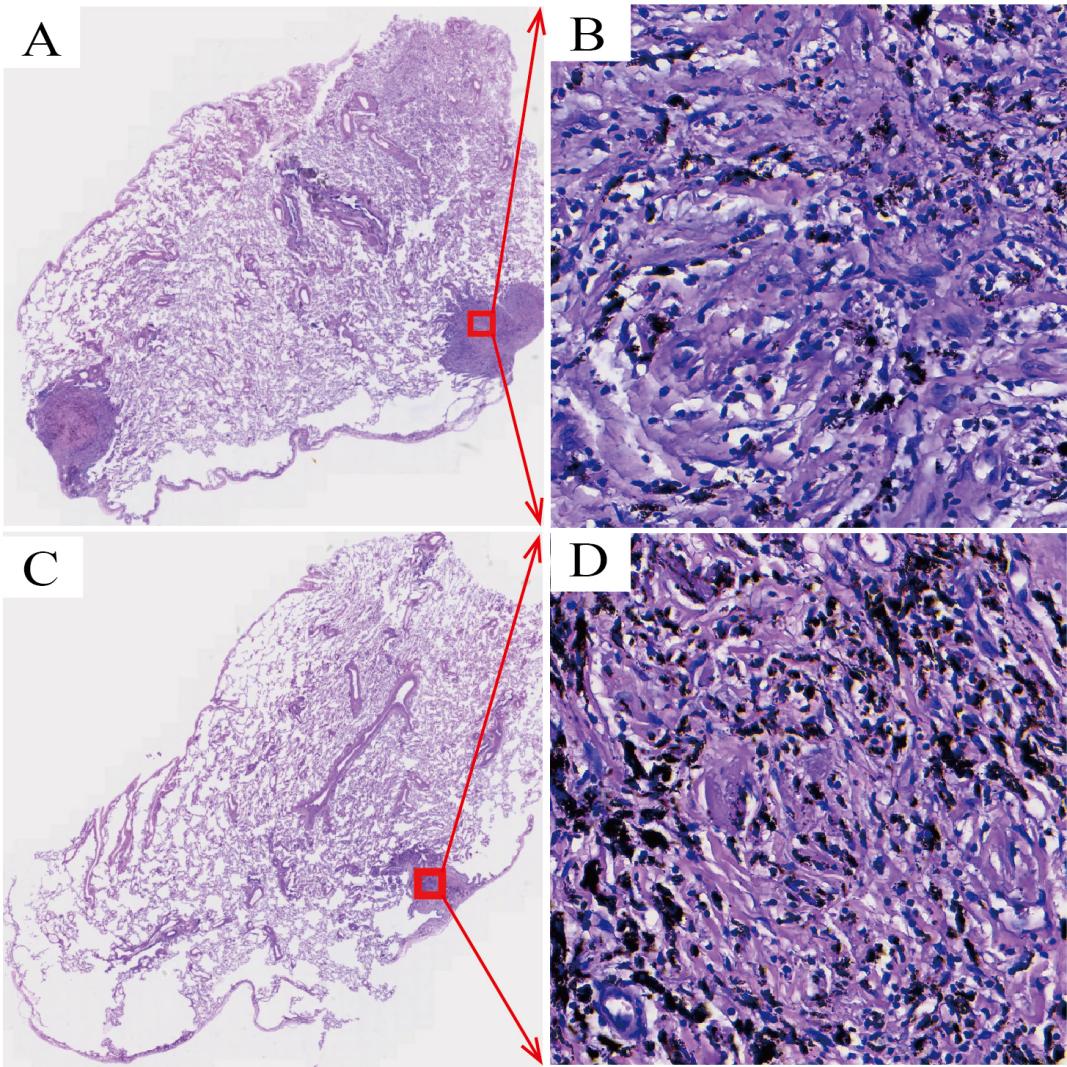


**Supplementary Fig. 1** (A, C). Overall the histopathology of benign nodules. (B,D). (HE-40X) Benign nodule biopsy showing proliferating spindle cells and collagen fibers.

*
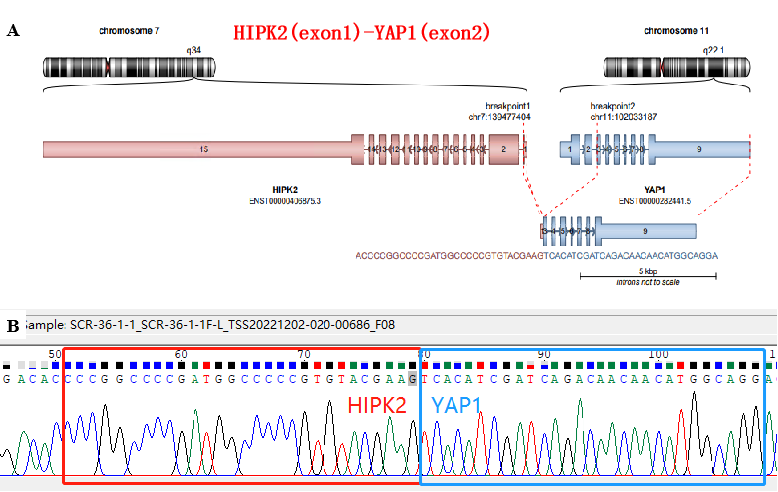
*

**Supplementary Fig. 2** HIPK2-YAP1 gene rearrangement (A) fusion mode; (B) sanger sequencing to validate fusion variants.
